# Provision of Care by “Real World” Telemental Health Providers

**DOI:** 10.3389/fpsyg.2021.653652

**Published:** 2021-05-07

**Authors:** Brian E. Bunnell, Nikolaos Kazantzis, Samantha R. Paige, Janelle Barrera, Rajvi N. Thakkar, Dylan Turner, Brandon M. Welch

**Affiliations:** ^1^Department of Psychiatry and Behavioral Neurosciences, Morsani College of Medicine, University of South Florida, Tampa, FL, United States; ^2^Doxy.me Research, Doxy.me, Inc., Rochester, NY United States; ^3^Cognitive Behavior Therapy Research Unit, Institute for Social Neuroscience Psychology, Melbourne, VIC, Australia; ^4^Biomedical Informatics Center, College of Medicine, Medical University of South Carolina, Charleston, SC, United States

**Keywords:** telemedicine, technology, mobile health, mental health, health delivery

## Abstract

Despite its effectiveness, limited research has examined the provision of telemental health (TMH) and how practices may vary according to treatment paradigm. We surveyed 276 community mental health providers registered with a commercial telemedicine platform. Most providers reported primarily offering TMH services to adults with anxiety, depression, and trauma-and stressor-related disorders in individual therapy formats. Approximately 82% of TMH providers reported endorsing the use of Cognitive Behavioral Therapy (CBT) in their remote practice. The most commonly used in-session and between-session (i.e., homework) exercises included coping and emotion regulation, problem solving, mindfulness, interpersonal skills, relaxation, and modifying and addressing core beliefs. CBT TMH providers had a higher odds of using in-session and homework exercises and assigning them through postal mail, email or fax methods, as compared to non-CBT TMH providers. TMH providers, regardless of treatment paradigm, felt that assigning homework was neither easy nor difficult and they believed their patients were somewhat-to-moderately compliant to their assigned exercises. CBT TMH providers also collected clinical information from their patients more often than non-CBT TMH providers. They reported being less satisfied with their method, which was identified most often as paper-based surveys and forms. Overall, TMH providers employ evidence-based treatments to their patients remotely, with CBT TMH providers most likely to do so. Findings highlight the need for innovative solutions to improve how TMH providers that endorse following the CBT treatment paradigm remotely assign homework and collect clinical data to increase their satisfaction via telemedicine.

## Introduction

Mental health disorders affect approximately 20% of adults and up to 50% of youth in the United States (U.S.), and less than half of these individuals will receive mental health services in a given year (Merikangas et al., 2010; SAMHSA, 2020). Younger adults, racial/ethnic minorities, men, and individuals residing in rural geographic regions experience disparities in health service utilization (Kirby et al., 2019). According to the Office of Disease Prevention and Health Promotion (2020), expanding treatment accessibility and utilization to medically underserved individuals with mental illness has been a national priority for over 10 years. This priority has strengthened in the recent year due to significant increases in anxiety and depression as a result of the COVID-19 pandemic (Holingue et al., 2020). Ongoing efforts to identify and optimize novel mental health care delivery programs, including who they best serve and how mental health services are delivered and evaluated, are important for promoting the public’s mental health.

Mental health care is a leading medical specialty in which providers use telemedicine to engage with their patients and deliver evidence-based treatments (Robeznieks, 2019). As a result, telemedicine has revolutionized the provision of mental health care (telemental health care or referred to hereinafter as TMH) worldwide by bringing accessible and personalized treatment to patients, families, and caregivers at a distance (Sable et al., 2002; Misra et al., 2005; Doolittle and Spaulding, 2006; Griffiths et al., 2006; Darkins et al., 2008; Lee and Park, 2016). The technology behind TMH is largely attributed to programs that host two-way transactions between patients and their providers (Breen and Matusitz, 2010). This includes real-time videoconferencing with providers to communicate about a diagnosis or remotely monitor an individual’s health status and progress related to care (Smith, 2007). TMH has a high degree of acceptability among both patients and providers (Gershkovich et al., 2016; Polinski et al., 2016; Kruse et al., 2017; Bunnell et al., 2020a), and it has been deemed an effective healthcare delivery solution that yields similar results as in-person treatment (Hilty et al., 2013; Langarizadeh et al., 2017; Veazie et al., 2019). Digital interventions have been especially useful to support widespread mental health promotion during the COVID-19 pandemic (Rauschenberg et al., 2021). In addition to its effectiveness, telemedicine transcends socio-cultural (i.e., stigma of mental illness and treatment) and geographic (i.e., limited transportation, remote location with shortages of medical professionals) barriers that have traditionally impeded patients’ access to and use of mental health services (Rojas and Gagnon, 2008; Baker et al., 2011; Wootton et al., 2011; Thaker et al., 2013).

Telemental health providers may believe that telemedicine is best suited for particular patients according to their demographic (e.g., age) and diagnosed mental health condition(s). In Gershkovich et al. (2016) study of 213 behavioral health providers, TMH was recommended for use among patients with depression and generalized anxiety. In another study, Simms et al. (2011) surveyed 160 and interviewed 25 mental health providers, who recommended against the use of TMH for patients who are emotionally unstable, impulsive, exhibit poor coping skills, and are clinically depressed or experiencing paranoia or a psychotic episode. Considering these discrepancies, surveillance of how TMH is used to treat various conditions (e.g., anxiety disorders and mood disorders) is needed. Generalizations that telemedicine is used equally to deliver treatment across all mental health conditions poses a risk for ill-informed policy recommendations that perpetuate limited access for vulnerable patient groups.

Cognitive behavioral therapy (CBT) is a gold-standard, evidence-based mental health treatment approach to alleviate symptoms of anxiety and depression. CBT is a skills-based psychotherapy focused on changing maladaptive thoughts and behaviors (David et al., 2018). During CBT, providers introduce and practice therapeutic exercises with patients in session and assign patients to practice those exercises between sessions (i.e., homework). Some common in-session CBT exercises delivered via TMH that have been examined in prior literature include self-monitoring (Morland et al., 2010; Lichstein et al., 2013; Hobbs et al., 2018), exposure therapy (Choi et al., 2014; Acierno et al., 2016; Hobbs et al., 2018), coping and emotion regulation (Morland et al., 2010; Choi et al., 2014), problem solving (Choi et al., 2014; Hobbs et al., 2018), and motivational strategies (Ruskin et al., 2004; Liebmann et al., 2019) that support behavioral activation (Lichstein et al., 2013; Acierno et al., 2016), mindfulness (Lichstein et al., 2013), interpersonal skills (Owen, 2019), relaxation (Lichstein et al., 2013), and self-management (Ruskin et al., 2004) therapies. Currently, there is little understanding of the use of these CBT exercises within real-world, TMH sessions (Mehrotra et al., 2016; Mehrotra et al., 2017; Shi et al., 2019).

Between-session, or homework exercises also are a crucial component of high-quality mental health care (Beck et al., 1979; Kazantzis and L’Abate, 2007). In essence, homework involves practicing in-session skills outside of therapy to master the behavior in a naturalistic setting. Kazantzis et al. (2000) found that assigning homework facilitates improvements in therapeutic outcomes, which is strengthened when patients comply with the assignments. However, prior studies have demonstrated that providers do not always follow recommended, best practice procedures for homework administration; that is, following guidelines for the review, design, and planning of homework (Kazantzis and Deane, 1999; Kazantzis et al., 2005a, b; Dattilio et al., 2011; Bunnell et al., 2020b). Moreover, patient homework adherence/compliance tends to be low-to-moderate (Helbig and Fehm, 2004; Gaynor et al., 2006; Bunnell et al., 2020b). Little research has examined the effectiveness of engaging patients in homework through online intervention (Tang and Kreindler, 2017); rather, homework has been predominantly studied within offline contexts. To begin building this body of literature, there is a need to explore how TMH providers engage their patients in homework to support treatment efforts.

With substantial causal and correlational effects of homework (Mausbach et al., 2010; Kazantzis et al., 2016), mental health professionals from a range of treatment paradigms report that their therapeutic practices are improved when they assign homework to patients (Kazantzis et al., 2005a). However, mental health providers who adopt CBT treatment paradigms are more likely to rate homework as “very important,” as compared to non-CBT endorsing providers who rate homework as “somewhat” or “moderately” important (Kazantzis and Dattilio, 2010). As a result, homework is most commonly used in cognitive and behavioral treatment paradigms, with scarce evidence for its use and effectiveness in alternate treatment paradigms (Kazantzis et al., 2000). It can be purported that TMH providers who endorse following a CBT treatment paradigm will be most likely to implement homework activities into their remote, therapeutic practices, as well. With empirical evidence needed to confirm the aforementioned claim, further research should be conducted to identify which types of homework exercises and methods for their assignment and assessment are used by CBT and non-CBT TMH providers.

Mental health providers are gatekeepers to telemedicine (Whitten and Mackert, 2005; Jameson et al., 2011; Cowan et al., 2019). They must see value in using telemedicine to deliver clinical care in order to foster its sustained uptake and implementation (Yuen et al., 2012). Exploring how TMH providers use telemedicine will be imperative for gauging its perceived value and optimizing its implementation during and after the COVID-19 pandemic, which has had a significant impact on the public’s mental health (Holingue et al., 2020). Conducting formative research to understand how “real world” TMH providers engage their patients and provide care will give insight to novel methods that optimize the implementation of telemedicine once the COVID-19 pandemic has ended (Stetler et al., 2006). Specifically, understanding how TMH care is provided in community practice settings will support a better understanding of the current needs of patients and providers and inform innovative solutions to meet their needs.

Thus, the purpose of the current study was to examine the provision of TMH practices among community mental health providers who do and do not endorse following the CBT treatment paradigm. We hypothesized that endorsing CBT as a treatment paradigm would be associated with the use of CBT-based in- and between-session exercises (e.g., problem solving, emotional and coping regulation) during TMH practice. With little research examining the administration of between-session exercises within remote care contexts, we explored differences in the frequency and methods used to assign between-session exercises and collect clinical data among providers depending on whether they endorse following the CBT treatment paradigm. Given the importance of patient homework compliance in promoting therapeutic outcomes (Kazantzis et al., 2000), we also examined how perceived patient compliance of between-session exercises varies according to whether or not the provider endorses following the CBT treatment paradigm.

## Materials and Methods

### Sample and Procedures

This is a report on a retrospective review of de-identified data from a web-based marketing survey administered among practicing mental health providers registered with doxy.me^1^, a commercial, HIPAA-compliant telemedicine platform with over 700,000 registered healthcare provider users, 40% of whom are mental health providers. According to a recent study (Bunnell et al., 2020a), doxy.me mental health providers are, on average, 46.4 years old (*SD* = 12.2; range = 26–83 years). They have been providing mental health care for 13.6 years (*SD* = 10.3; range = 1–49 years). The majority of providers are female (69.1%), white (78.5%), and non-Hispanic (91.5%). These providers are primarily licensed mental health counselors (33%), psychologists (22.2%), or social workers (21%) with masters or doctoral-level education. They are either employed in an independent practice (70.1%) or part of a small clinic (18.1%).

We invited mental health providers registered with doxy.me to participate in a survey, which was conducted between September 17, 2019 and October 31, 2019. English speaking, licensed mental and behavioral health providers registered with the doxy.me telemedicine platform were included. Of the 1,910 providers who were sent email invitations, 349 (18.3%) responded and 276 (15.0%) completed the survey, a completion rate of 79.1%. The average survey completion time was 18 min. The survey began with information about the purpose of the data collection–to gain insight into their use of telemedicine for mental health services and to improve their doxy.me experience. Next, providers were asked to consent to have their de-identified data used for marketing research and possible publication. Compensation was not offered, and the Institutional Review Board at the University of South Florida determined that this retrospective review was human subjects exempt.

### Survey and Measures

The survey was developed by the Doxy.me, LLC marketing and leadership teams based on their knowledge and experience in the field of telemedicine, consultations with an experienced TMH provider, and prior survey studies on telemedicine and aspects of mental health treatment (Kazantzis and Deane, 1999; Kazantzis et al., 2005a; Kazantzis and Dattilio, 2010; Welch et al., 2016; Bunnell et al., 2019). Item readability and relevance and survey efficiency were assessed through an iterative review process.

The survey began with questions about providers’ typical TMH caseloads (i.e., age groups and mental health conditions treated) and treatment approaches (i.e., therapy formats and treatment orientation). Next, providers were asked to indicate which CBT exercises they routinely conduct in-session with their TMH patients, and which of those, if any, are too difficult to conduct via telemedicine. Providers were then asked to indicate how often they assign between-session CBT exercises to their TMH patients (i.e., 1 = “Never” to 5 = “Almost Always”), which between-session exercises they routinely assign, how compliant their TMH patients are to those assignments (i.e., 1 = “Not Very Compliant” to 4 = “Highly Compliant”), the methods they use to assign and assess between-session exercises (e.g., verbal interview, electronic or paper surveys) and how easy/difficult it is for them to assign/assess (i.e., 1 = “Very Easy” to 5 = “Very Difficult”). Lastly, providers were asked to indicate how frequently they collect clinical data from their patients (i.e., 1 = “Never” to 5 = “Almost Always”), the methods they use for data collection (e.g., paper or electronic surveys/forms, verbal interview) and their satisfaction with their current method of collecting patient data (i.e., 1 = “Very Dissatisfied” to 5 = “Very Satisfied”).

### Data Analysis

SPSS v26 (IBM Corp.) was used to compute frequency and descriptive statistics on provider caseload and treatment approaches used by TMH providers. We computed frequency and descriptive statistics on the prevalence and method of assigning in-session and between-session exercises, as well as their relative difficulty to conduct these exercises on a telemedicine platform. Finally, we computed frequency and descriptive statistics to report on how clinical data are collected during consultations. Providers were categorized according to whether or not they endorsed following the CBT treatment paradigm, where 1 = CBT (e.g., CBT, behavioral, and social learning) and 0 = non-CBT (e.g., psychodynamic, interpersonal, and existential/humanistic). We computed a series of logistic regression analyses to determine if the frequency and methods for assigning in-session and between session-exercises were most common among TMH providers who endorsed following the CBT treatment paradigm. We also conducted a series of independent samples t-tests to examine how aspects of assigning between-session exercises, perceiving patient compliance to assigned homework assignments, and collecting clinical data varied among TMH providers who did and did not endorse following the CBT treatment paradigm.

## Results

### Telemental Health Caseload and Treatment Approaches

Data relating to provider caseloads and treatment approaches are displayed in Table 1. Almost all providers (*n* = 272; 98.6%) reported regularly providing TMH services to adults (18–64 years old), whereas less than 20% of providers reported regularly providing TMH services to adolescents (11–17 years old), older adults (65+ years old), and children (0–10 years old). The vast majority of TMH providers reported primarily treating anxiety (*n* = 261; 94.6%), mood (*n* = 240; 87.0%), and trauma-and stress-related disorders (*n* = 213; 77.2%). Less than half (*n* = 115; 41.7%) of providers reported primarily treating obsessive-compulsive disorders. Personality, impulse-control, somatic, food/eating, sexual dysfunction, dissociative, and gender dysmorphia disorders were treated primarily by less than 30% of providers.

**TABLE 1:** Characteristics of telemental health caseload and treatment approaches.

**Characteristic**	***n***	**%**
**Age groups seen**		
Adults (18–64 years old)	272	98.6
Adolescents (11–17 years old)	45	16.3
Older adults (65+ years old)	33	12.0
Children (0–10 years old)	10	3.6
**Mental health conditions treated**		
Anxiety disorders	261	94.6
Mood disorders	240	87.0
Trauma- and stressor-related disorders	213	77.2
Obsessive-compulsive and related disorders	115	41.7
Substance-related and addictive disorders	78	28.3
Personality disorders	65	23.6
Disruptive, impulse-control, and conduct disorders	57	20.7
Somatic symptom and related disorders	54	19.6
Gender dysphoria	50	18.1
Dissociative disorders	47	17.0
Sexual dysfunctions	38	13.8
Feeding and eating disorders	28	10.1
Neurocognitive disorders	19	6.9
Neurodevelopmental disorders	17	6.2
Schizophrenia spectrum and other psychotic disorders	13	4.7
Paraphilic disorders	6	2.2
**Therapy formats typically used**		
Individual therapy	273	98.9
Couples therapy	59	21.4
Family therapy	24	8.7
Group therapy	4	1.4
**Treatment paradigms typically used**		
Cognitive-behavioral	226	81.9
Interpersonal	141	51.1
Family systems	121	43.8
Existential/humanistic	117	42.4
Psychodynamic/analytic	104	37.7
Behavioral	90	32.6
Social learning	45	16.3

*Providers were asked to select “all that apply” for each item. Categories are not mutually exclusive, and columns do not sum 100%.*

Nearly all (*n* = 272; 98.9%) TMH providers reported conducting individual therapy, while only a small percentage reported providing couples (*n* = 59; 21.4%), family (*n* = 24; 8.7%), and group (*n* = 4; 1.4%) therapy via telemedicine. When asked about which treatment paradigms they typically use, the majority of providers endorsed following the CBT paradigm (*n* = 226; 81.9%), with the next most commonly used treatment paradigms including interpersonal (*n* = 141; 51.1%), family systems (*n* = 121; 43.8%), existential/humanistic (*n* = 117; 42.4%), psychodynamic/analytic (*n* = 104; 37.7%), and behavioral (*n* = 90; 32.6%).

### Conducting In-Session Exercises With Telemental Health Patients

Data relating to conducting in-session exercises are displayed in Table 2. The most common in-session exercises that providers reported routinely conducting with their TMH patients included coping/emotion regulation (*n* = 240; 87.0%), problem solving (*n* = 225; 81.5%), mindfulness (*n* = 218; 79.0%), interpersonal skills (*n* = 207; 75.0%), and relaxation (*n* = 207; 75.0%). The least common in-session exercises routinely conducted by providers included arousal reduction/management (*n* = 61; 22.1%), attentional flexibility (*n* = 42; 15.2%), contingency management (*n* = 37; 13.4%), stimulus control (*n* = 29; 10.5%), and shaping (*n* = 25; 9.1%). About half of providers (*n* = 130; 47.1%) indicated they did not find any in-session exercises too difficult to conduct via telemedicine. Exposure strategies and crisis management were identified as too difficult to conduct via telemedicine by roughly 15% of providers. Perceptions about the difficulty of conducting in-session exercises did not vary among CBT and non-CBT TMH providers based on logistic regression analysis results.

**TABLE 2:** Conducting in-session exercises with telemental health patients.

**Type of exercise**	**Total *n* (%)**	**Providers endorsing in-session exercises**	
		**CBT *n* (%)**	**Non-CBT *n* (%)**
**Routinely conducted in-session exercises**			
Coping and emotion regulation	240 (87.0)	212 (88.7)	27 (11.3)
Problem solving	225 (81.5)	198 (89.2)	24 (10.8)
Mindfulness	218 (79.0)	191 (88.0)	26 (12.0)
Interpersonal skills	207 (75.0)	179 (86.9)	27 (13.1)
Relaxation (e.g., deep breathing)	207 (75.0)	179 (87.3)	26 (12.7)
Modifying and addressing core beliefs	197 (71.4)	176 (89.8)	20 (10.2)
Motivational strategies	176 (63.8)	157 (90.2)	17 (9.8)
Cognitive flexibility and reappraisal	169 (61.2)	158 (93.5)	11 (6.5)
Self-monitoring	145 (52.5)	128 (88.3)	17 (11.7)
Psychological acceptance	134 (48.6)	119 (88.8)	15 (11.2)
Self-management	130 (47.1)	115 (89.1)	14 (10.9)
Values	128 (46.4)	116 (91.3)	11 (8.7)
Crisis management	128 (46.4)	117 (92.1)	10 (7.9)
Defusing/distancing	101 (36.9)	92 (91.1)	9 (8.9)
Behavioral activation	81 (29.3)	77 (95.1)	4 (4.9)
Exposure strategies	73 (26.4)	67 (91.8)	6 (8.2)
Arousal reduction or management	61 (22.1)	53 (86.9)	8 (13.1)
Attentional flexibility	42 (15.2)	38 (90.5)	4 (9.5)
Contingency management	37 (13.4)	35 (94.6)	2 (5.4)
Stimulus control	29 (10.5)	28 (96.6)	1 (3.4)
Shaping	25 (9.1)	23 (92.0)	2 (8.0)
NONE	2 (0.7)	0 (0.0)	2 (100.0)
**In-session exercises too difficult to conduct**			
NONE	130 (47.1)	110 (86.6)	17 (13.4)
Exposure strategies	44 (15.9)	38 (88.4)	5 (11.6)
Crisis management	39 (14.1)	31 (79.5)	8 (20.5)
Stimulus control	19 (6.9)	18 (94.7)	1 (5.3)
Arousal reduction or management	19 (6.9)	14 (73.7)	5 (26.3)
Relaxation (e.g., deep breathing)	18 (6.5)	17 (94.4)	1 (5.6)
Mindfulness	14 (5.1)	13 (92.9)	1 (7.1)
Behavioral activation	14 (5.1)	12 (85.7)	2 (14.3)
Defusing/distancing	10 (3.6)	8 (80.0)	2 (20.0)
Shaping	7 (2.5)	6 (85.7)	1 (14.3)
Attentional flexibility	7 (2.5)	4 (57.1)	3 (42.9)
Interpersonal skills	6 (2.2)	4 (66.7)	2 (33.3)
Self-monitoring	6 (2.2)	4 (66.7)	2 (33.3)
Coping and emotion regulation	4 (1.4)	3 (75.0)	1 (25.0)
Cognitive flexibility and reappraisal	3 (1.1)	3 (100.0)	0 (0.0)
Contingency management	3 (1.1)	2 (66.7)	1 (33.3)
Self-management	3 (1.1)	2 (66.7)	1 (33.3)
Values	3 (1.1)	3 (100.0)	0 (0.0)
Modifying and addressing core beliefs	1 (0.4)	1 (100.0)	0 (0.0)
Motivational Strategies	1 (0.4)	1 (100.0)	0 (0.0)
Problem solving	1 (0.4)	1 (100.0)	0 (0.0)
Psychological acceptance	1 (0.4)	1 (100.0)	0 (0.0)

*Providers were asked to select “all that apply” for each item; therefore, categories are not mutually exclusive, and columns do not sum 100%. The frequency of in-session exercises in the “total” column may not equal the sum of corresponding rows capturing providers’ use according to CBT vs. non-CBT endorsement due to missing data points.*

Logistic regression analyses demonstrated that CBT TMH providers had significantly higher odds of using certain in-session exercises than non-CBT TMH providers. These included behavioral activation (OR = 4.38, 95% Confidence Interval [CI] = 1.5–12.76; *p* < 0.01), cognitive flexibility (OR = 5.51; 95% CI = 2.60–11.67; *p* < 0.001), coping/emotion regulation (OR = 4.96; 95% CI = 2.17–11.33; *p* < 0.001), crisis management (OR = 2.98; 95% CI = 1.39–6.39; *p* = 0.005), mindfulness (OR = 2.39; 95% CI = 1.13–5.04; *p* < 0.05), modifying and addressing core beliefs (OR = 3.04; 95% CI = 1.51–6.10; *p* < 0.01), motivational strategies (OR = 2.75; 95% CI = 1.38–5.48; *p* < 0.01), problem solving (OR = 3.75; 95% CI = 1.78–7.88; *p* < 0.001), and values clarification (OR = 2.57; 95% CI = 1.22–5.40; *p* < 0.05).

### Assigning and Assessing Homework With Telemental Health Patients

Data relating to assigning and assessing homework are displayed in Table 3. The most common homework exercises that providers routinely assigned to their TMH patients included mindfulness (*n* = 210; 76.1%), coping and emotional regulation (*n* = 191; 69.2%), and relaxation (*n* = 189; 68.5%). The least common homework exercises routinely assigned by providers included arousal reduction or management (*n* = 56; 20.3%), crisis management (*n* = 50; 18.1%), attentional flexibility (*n* = 43; 15.6%), contingency management (*n* = 34; 12.3%), stimulus control (*n* = 26; 9.4%), and shaping (*n* = 16; 5.8%).

**TABLE 3:** Assigning and assessing homework exercises with telemental health patients.

**Between-session variables**	**Total *n* (%)**	**Providers endorsing homework exercises**	
		**CBT *n* (%)**	**Non-CBT *n* (%)**
**Routinely conducted homework exercises^t^**			
Mindfulness	210 (76.1)	24 (11.5)	185 (88.5)
Coping and emotion regulation	191 (69.2)	173 (90.6)	18 (9.4)
Relaxation (e.g., deep breathing)	189 (68.5)	166 (88.3)	22 (11.7)
Self-monitoring	150 (54.3)	133 (89.9)	15 (10.1)
Interpersonal skills	148 (53.6)	131 (89.7)	15 (10.3)
Problem solving	143 (51.8)	133 (93.7)	9 (6.3)
Cognitive flexibility and reappraisal	132 (47.8)	127 (96.9)	4 (3.1)
Self-management	110 (39.9)	100 (91.7)	9 (8.3)
Modifying and addressing core beliefs	104 (37.7)	97 (93.3)	7 (6.7)
Behavioral activation	94 (34.1)	88 (94.6)	5 (5.4)
Exposure strategies	79 (28.6)	76 (96.2)	3 (3.8)
Defusing/distancing	69 (25.0)	65 (94.2)	4 (5.8)
Motivational strategies	66 (23.9)	61 (93.8)	4 (6.2)
Psychological acceptance	63 (22.8)	59 (93.7)	4 (6.3)
Arousal reduction or management	56 (20.3)	51 (91.1)	5 (8.9)
Values	54 (19.6)	49 (92.5)	4 (7.5)
Crisis management	50 (18.1)	47 (94.0)	3 (6.0)
Attentional flexibility	43 (15.6)	38 (88.4)	5 (11.6)
Contingency management	34 (12.3)	33 (100.0)	0 (0.0)
Stimulus control	26 (9.4)	25 (96.2)	1 (3.8)
Shaping	16 (5.8)	16 (100.0)	0 (0.0)
**Methods for assigning homework to patients^t^**			
Explain verbally during a session	235 (86.4)	203 (87.9)	27 (12.1)
Send a resource via postal mail, email, fax	125 (46.0)	114 (91.9)	10 (8.1)
Provide a link to download materials	73 (26.8)	68 (93.2)	5 (6.8)
Provide materials in person	38 (14.0)	37 (92.5)	3 (7.5)
Send by text message	40 (14.7)	35 (92.1)	3 (7.9)
Use a 3rd party software for a specific treatment	13 (4.8)	12 (100.0)	0 (0.0)
Use telemedicine feature (e.g., screen share)	7 (2.6)	7 (100.0)	0 (0.0)
Use a web-based survey (e.g., SurveyMonkey)	3 (1.1)	3 (100.0)	0 (0.0)

*^t^ = providers were asked to select “all that apply.” Categories are not mutually exclusive, and columns do not sum 100%. The frequency of using and assigning between-session exercises in the “total” column may not equal the sum of corresponding rows capturing providers’ use by CBT vs. non-CBT endorsement due to missing data points.*

Similar to in-session exercises, logistic regression analyses demonstrated that CBT TMH providers had significantly higher odds of using certain between-session exercises than non-CBT providers. These included behavioral activation (OR = 4.19; 95% CI = 1.58–11.10; *p* < 0.01), cognitive flexibility (OR = 10.69; 95% CI = 3.68–31.04; *p* < 0.001), coping/emotion regulation (OR = 1.25; 1.74–6.98; *p* < 0.001), defusing and distancing (OR = 3.43; 95% CI = 1.17–10.02; *p* < 0.05), exposure strategies (OR = 5.88; 95% CI = 1.76–19.72; *p* < 0.01), interpersonal skills (OR = 2.10; 95% CI = 1.05–4.20; *p* < 0.05), mindfulness (OR = 2.52; 95% CI = 1.22–5.17; *p* < 0.01), modifying and addressing core beliefs (OR = 3.31; 95% CI = 1.40–7.81; *p* < 0.01), motivational strategies (OR = 3.14; 95% CI = 1.07–9.20; *p* < 0.05), problem solving (OR = 4.52; 95% CI = 2.06–9.96; *p* < 0.001), self-management (OR = 2.55; 95% CI = 1.16–5.60; *p* < 0.05), and self-monitoring (OR = 2.17; 95% CI = 1.08–4.36; *p* < 0.05).

Table 4 shows that, on average, providers reported that they assigned homework to their TMH patients “Sometimes” to “Often” (*M* = 3.76, *SD* = 0.99). CBT TMH providers mostly reported assigning homework “Often” (*M* = 3.90, *SD* = 0.91), whereas non-CBT providers reported using homework assignments in their practice only “Sometimes” (*M* = 3.00, *SD* = 1.10; *t*[268] = −5.53, *p* < 0.001, Cohen’s *d* = 0.96). CBT and non-CBT TMH providers reported that their patients were “Somewhat” to “Moderately” compliant to homework assignments (*M* = 2.78, *SD* = 0.69). They also reported that assigning and assessing homework with their TMH patients was “neither easy nor difficult” (*M* = 3.36, *SD* = 0.83).

**TABLE 4:** Process of assigning homework via telemedicine.

**Between-session assignment variables**	**Total *n* (%)**	**Treatment paradigm**	
		**CBT *n* (%)**	**Non-CBT *n* (%)**
**Frequency of assigning homework exercises**			
Almost always	75 (27.3)	72 (31.2)	3 (7.7)
Often	89 (32.4)	76 (32.9)	11 (28.2)
Sometimes	84 (30.5)	72 (31.2)	11 (28.2)
Rarely	23 (8.4)	10 (4.3)	11 (28.2)
Never	4 (1.4)	1 (0.4)	3 (7.7)
**Difficulty assigning and assessing homework remotely**			
Very easy	32 (12.0)	24 (10.5)	6 (17.6)
Easy	57 (21.3)	51 (22.4)	6 (17.6)
Neither easy nor difficult	156 (58.4)	134 (58.8)	20 (58.8)
Difficult	19 (7.1)	16 (7.0)	2 (5.9)
Very difficult	3 (1.1)	3 (1.3)	0 (0.0)
**TMH patient compliance to homework assignments**			
Highly compliant	40 (14.7)	33 (14.3)	7 (19.4)
Moderately compliant	135 (49.6)	116 (50.4)	14 (38.9)
Somewhat compliant	95 (34.9)	80 (34.8)	15 (41.7)
Not very compliant	2 (0.7)	1 (0.4)	0 (0.0)

*The frequency of response options in the “total” column may not equal the sum of corresponding rows capturing providers’ use by CBT vs. non-CBT endorsement due to missing data points.*

Most providers assigned homework exercises to their TMH patients verbally during sessions (*n* = 235; 86.4%); however, almost half of providers reported that they sent homework materials via postal mail, email, or fax (*n* = 125; 46.0%) or provided links to download materials (*n* = 73; 26.8%). Less common methods used to assign homework included providing materials in person (*n* = 40; 14.7%) and sending text messages (*n* = 38; 14.0%). Logistic regression analyses demonstrated that CBT TMH providers had a significantly higher likelihood of assigning homework via postal mail, email, or fax than non-CBT providers (OR = 2.55; 95% CI = 1.18–5.54; *p* < 0.05), and a marginally significantly higher likelihood of assigning homework by providing a link to download materials (OR = 2.60; 95% CI = 0.97–6.98; *p* = 0.06) or explaining verbally during a session (OR = 2.15; 95% CI = 0.89–5.19; *p* = 0.09).

### Collecting Clinical Data From Telemedicine Consultations

Data relating to collecting clinical data from patients are displayed in Table 5. On average, providers reported that they collected clinical data from their TMH patients “Sometimes” to “Often” (*M* = 3.65; *SD* = 1.03). Two thirds of providers reported using verbal interview methods to collect these data (*n* = 184; 66.7%). Other common methods included paper (*n* = 142; 51.4%) and electronic (*n* = 115; 41.7%) surveys or forms.

**TABLE 5:** Collecting clinical data from telemedicine patients.

**Data collection variables**		**Treatment paradigm**	
	**Total *n* (%)**	**CBT *n* (%)**	**Non-CBT *n* (%)**
**Frequency of collecting clinical data from patients**			
Almost always	70 (25.8)	59 (26.0)	7 (17.9)
Often	76 (28.0)	70 (30.8)	6 (15.4)
Sometimes	88 (32.5)	77 (33.9)	11 (28.2)
Rarely	34 (12.5)	20 (8.8)	13 (33.3)
Never	3 (1.1)	1 (0.4)	2 (5.1)
**Method for collecting clinical data from patients^t^**			
Verbal interview	184 (66.7)	154 (83.7)	30 (16.3)
Paper survey or form	142 (51.4)	127 (91.4)	12 (8.6)
Electronic survey or form	115 (41.7)	96 (85.7)	16 (14.3)
**Satisfaction with clinical data collection method**			
Very satisfied	66 (24.3)	49 (21.4)	17 (44.7)
Satisfied	116 (42.6)	96 (41.9)	16 (42.1)
Neither satisfied nor dissatisfied	68 (25.0)	64 (27.9)	3 (7.9)
Dissatisfied	22 (8.1)	20 (8.7)	2 (5.3)
Very dissatisfied	0 (0.0)	0 (0.0)	0 (0.0)

*^t^ = Providers were asked to select “all that apply”; therefore, categories are not mutually exclusive, and columns do not sum 100%. Also, the frequency of response options in the “total” column may not equal the sum of corresponding rows capturing providers’ use by CBT vs. non-CBT endorsement due to missing data points.*

On average, CBT TMH providers (*M* = 3.73, *SD* = 0.88) reported collecting information from their patients more often than non-CBT providers (*M* = 3.08, *SD* = 1.20; *t*[264] = −3.78, *p* < 0.001, Cohen’s *d* = 0.65). A subsequent analysis found that CBT TMH providers were less satisfied (*M* = 3.75, *SD* = 0.89) with their current method of collecting information about their patients than non-CBT providers (*M* = 4.26, *SD* = 0.83; *t*[265] = 3.27, *p* < 0.001, Cohen’s *d* = 0.57). A logistic regression analysis demonstrated that CBT TMH providers had a significantly higher likelihood of collecting data via paper-based surveys or forms than non-CBT providers (OR = 2.75; 95% CI = 1.33–5.69; *p* < 0.01).

## Discussion

The purpose of this study was to investigate how TMH care is provided in community practice settings to gain a better understanding of TMH providers, their patients, and their provision of remote care. We found that TMH providers who endorse following the CBT paradigm are more likely to use evidence-based in-session and between-session (i.e., homework) exercises in their practice, including (but not limited to) behavioral activation, cognitive flexibility, and coping and emotional regulation. These providers report assigning homework to their patients more frequently and have a greater likelihood of assigning homework through postal mail, email, or fax compared to non-CBT providers. TMH providers who endorsed following the CBT treatment paradigm also collected clinical data from their patients more frequently despite reporting less satisfaction with their collection method, which was most commonly using paper-based surveys or forms. In addition to providing insight for using telemedicine to deliver CBT, this report offers important implications for telemedicine to mitigate negative health outcomes during the COVID-19 pandemic.

### Principal Findings

Individual therapy was practiced among nearly all TMH providers, with over half of providers reporting that they treat adults with anxiety, mood, and trauma- and stressor-related disorders. Anxiety and mood-related disorders are the most prevalent psychiatric disorders (Bandelow and Michaelis, 2015), and are commonly treated in community settings via telemedicine (Gershkovich et al., 2016). Although TMH providers in the current study reported treating individuals across the lifespan, they most commonly provided services to adults between the ages of 18 and 64. This finding is promising given that mental health promotion is beneficial at any age (Cattan and Tilford, 2006). In 2017, adults 18–25 years old had the highest past year prevalence of having any mental illness (25.8%), yet they were less likely to have received treatment compared to adults over the age of 25 (SAMHSA, 2020). Generations are not homogenous in regard to values, preferences, and needs for healthcare delivery and related experiences. Therefore, efforts are needed to understand the best ways to initiate and engage patients in evidence-based TMH for anxiety and mood-related disorders across the lifespan.

Most TMH providers reported following the CBT treatment paradigm in their remote practices. CBT is among the most effective psychotherapy paradigms and is considered the gold standard treatment for anxiety and related disorders (David et al., 2018). CBT TMH providers had greater odds of using a constellation of evidence-based in-session activities with their patients remotely, including (but not limited to) cognitive flexibility, coping and emotional regulation, and behavioral activation. Using telemedicine to deliver CBT has demonstrated a high degree of reach and patient satisfaction, as well as improvements in mental health-related outcomes (Dent et al., 2018). Half of all TMH providers indicated that none of the in-session exercises were too difficult to conduct via telemedicine. Among the TMH providers that selected at least one challenging in-session exercise to conduct remotely, about 15% selected exposure therapy and crisis management. Implementation strategies to overcome challenges with delivering in-session exercises via telemedicine are needed, especially for those who identify as CBT TMH providers. Measuring challenges with conducting in-session exercises with a dichotomous measure (e.g., “yes” vs. “no”) will gauge which exercises are the most challenging from an extremist perspective. However, examining perceived difficulties on a continuum (e.g., via Likert scale) will allow more variability in responses and provide insight to when and how to intervene.

A central component of high-quality, mental health treatment includes assigning homework, or practicing skills learned during treatment outside of the sessions with a provider (Kazantzis and Ronan, 2006). Homework is typically underutilized in mental health treatment (Kazantzis et al., 2005a, b; Dattilio et al., 2011; Bunnell et al., 2020b). Consistent with this knowledge, TMH providers who responded to this survey generally reported only sometimes assigning homework. When examining the use of between-session exercises according to treatment paradigm, CBT TMH providers reported more frequently assigning between-session exercises and assigning a greater variety of these exercises to their caseload than non-CBT TMH providers. Between-session exercises commonly assigned by CBT TMH providers included (but were not limited to) cognitive flexibility, coping and emotion regulation, exposure strategies, and behavioral activation. Consistent with evidence that between-session exercises are most commonly used by providers following CBT paradigms (Kazantzis et al., 2000), results of this study demonstrate that this evidence translates to community mental health providers who use telemedicine in their practice.

Providers in prior research have typically reported that patients demonstrate low-to-moderate homework compliance/adherence, and this is a commonly cited barrier to high quality mental health treatment (Helbig and Fehm, 2004; Gaynor et al., 2006; Bunnell et al., 2020b). TMH providers who completed this survey reported comparable compliance rates, despite reporting that they generally found assigning and assessing homework via telemedicine neither easy nor difficult. Patient compliance and perceived challenges in assessing homework did not differ among CBT *vs*. non-CBT TMH providers. While CBT TMH providers were most likely to use mail (e.g., postal, email, or fax) to assign homework, TMH providers in this study generally used a common method of verbally instructing patients how to conduct the homework. Patient compliance to homework is due to numerous factors and the assessment of compliance is multi-faceted, engaging the patient and avoiding a one-size-fits-all approach (Kazantzis et al., 2017). There is a need for innovative solutions to improve the assignment and assessment of homework via telemedicine, as the methods being used do not align with best practice recommendations (Kazantzis et al., 2005b).

Another affordance of telemedicine is the capability to collect and record clinical data from patients. Surveyed TMH providers reported collecting clinical data from their patients most often through verbal interviews, followed by paper and electronic surveys. Further, providers indicated feeling very satisfied with their data collection methods. Verbal interviews are invaluable to establish patient-provider rapport and to gain rich data to guide clinical consultations and treatment decisions. However, alongside advancements in telemedicine functionality are the evolving systems that automate this process, making clinical data collection procedures more efficient and enhancing their precision for personalized care coordination. Should TMH providers prefer to continue collecting clinical data through forms of verbal interviews, data analysis and interpretation techniques must advance to meet the evolving nature of telemedicine. One example is leveraging partnerships with computer scientists to use machine learning techniques to process and distill clinical data in a reliable and clinically meaningful manner (Chen et al., 2018). This will be especially crucial for CBT TMH providers who report most frequently assigning homework and collecting clinical data from patients remotely via paper-based methods while indicating suboptimal satisfaction with the process.

### Limitations

This report has limitations that should be considered while interpreting results and coordinating future research. First, data were collected using a purposive sampling approach. There was not a designated sampling frame to ensure that a representative sample of TMH providers were recruited to complete the survey. Although this may raise concerns about the generalizability of results, it is notable that the socio-demographic characteristics of TMH providers registered with doxy.me is representative of the mental health workforce in the United States (American Psychological Association, 2015; Salsberg et al., 2017). A second limitation is that data are based upon self-reported data and may be prone to reporting bias. Likewise, patient compliance results were derived from data based on providers’ perceptions and not standardized or behavioral assessments. Another limitation is the low response rate (15%), although this is consistent with average response rates for web-based surveys (Cook et al., 2000). While efforts could have been made to promote responses such as reminder emails and follow-up phone calls and texts, this low response rate demonstrates a strong need for research delineating best practices for recruiting healthcare providers registered with telemedicine and other health technology platforms. Despite this, the completion rate of approximately 79% is a notable strength of this survey. With these limitations, it is important to keep in mind that this report includes a secondary analysis of de-identified marketing data from a commercial telemedicine company.

### Practical Implications

It is undeniable that telemedicine has expanded access to mental healthcare across the nation (Spivak et al., 2020). However, literature demonstrating the effectiveness of TMH treatment is comprised of studies conducted within and among providers who are employed by research-driven clinics (Hilty et al., 2013; Flodgren et al., 2015; Jenkins-Guarnieri et al., 2015; Langarizadeh et al., 2017; Adams et al., 2018). Patients treated in clinical research settings experience superior improvement in health outcomes as compared to their counterparts who receive treatment in non-research settings, even while controlling for therapist training history and the patient population (Gibbons et al., 2013). Findings of this report demonstrate that “real world,” community-based TMH providers do employ evidence-based therapeutic approaches that are empirically shown to improve health outcomes among patients.

To offer the highest quality TMH care to patients, there is a need to optimize how providers assign and engage patients in between-session homework assignments. Most providers use verbal or hard copy mailed instructional methods to assign and evaluate homework, whereas a small proportion of TMH providers used text messaging or third-party software programs. Reviews have demonstrated the efficiency, effectiveness, and satisfaction of text-based messages in promoting patient adherence in a variety of health contexts (Hall et al., 2015; Thakkar et al., 2016; Mayer and Fontelo, 2017). Echoing the recommendation of these reviews on the effectiveness of text-based messaging, there is a significant need to understand how the degree of message tailoring to the patient (e.g., personalization, feedback, and content matching), dosage or frequency of delivery, as well as the opportunities for TMH providers to customize the message influences their acceptance, feasibility, and effectiveness. In addition, formative research is needed to understand the motivations and barriers of TMH providers in using text message-based messages to facilitate homework assignments. This includes optimizing the accessibility of text-based features given the degree it is integrated into a telemedicine platform.

Although not the primary purpose of this report, the results have important implications for the delivery of TMH treatment during unprecedented times, such as the COVID-19 pandemic. Data in this report were collected prior to COVID-19 and may not necessarily generalize to the current state of mental health care practices among TMH providers, who commonly supplement in-person treatment with telemedicine sessions (Gershkovich et al., 2016). Since the onset of the COVID-19 pandemic, when social distancing mandates and regulatory guidelines hindering typical clinical interactions, there has been an exponential increase in healthcare providers who use telehealth services (Koonin et al., 2020). Understanding how these novice users are adapting to providing TMH services is an important area of inquiry that has potential to advance the quality of care conducted via telemedicine.

## Conclusion

Community-based mental TMH providers commonly deliver TMH services to adults with anxiety, depression, and trauma-and stress-related disorders in individual therapy formats and follow the CBT treatment paradigm. These sessions include evidence-based, in-session CBT exercises. Providers also report assigning exercises for patients to practice between sessions for homework. Despite moderate perceptions of patient compliance, CBT TMH providers report “often” assigning and assessing homework whereas non-CBT TMH providers only “sometimes” do so. Differences in how clinical data were collected from CBT and non-CBT TMH providers also emerged, with CBT TMH providers reporting collecting these data in paper-based forms and feeling low satisfaction with the process. Results highlight the need for more innovative solutions to improve the assignment and assessment of homework via telemedicine, as it has become a primary method of healthcare delivery since the COVID-19 pandemic.

## Data Availability Statement

The raw data supporting the conclusions of this article will be made available by the authors, without undue reservation.

## Ethics Statement

The studies involving human participants were reviewed and approved by University of South Florida IRB. The participants provided their written informed consent to participate in this study.

## Author Contributions

BW, NK, DT, and BB: conceptualization and methodology. SP and BB: formal analysis. BW and DT: investigation and resources. DT and BB: data curation. BB, JB, RT, NK, and SP: writing—original draft preparation. BB, JB, RT, BW, DT, NK, and SP: writing—review and editing. All authors have read and agreed to the published versionof the manuscript.

## Conflict of Interest

BW and DT are shareholders of Doxy.me, Inc. BB, SP, and JB are employed by Doxy.me, Inc. The remaining authors declare that the research was conducted in the absence of any commercial or financial relationships that could be construed as a potential conflict of interest.

## Footnotes

1 https://doxy.me/
